# Comparative Prognostic Evaluation of the Revised International Federation of Gynecology and Obstetrics 2023 and 2009 Staging Systems in Early Endometrial Cancer

**DOI:** 10.3390/cancers17183017

**Published:** 2025-09-16

**Authors:** Su Lim Lee, Yu Ri Shin, Hokun Kim, Sung Eun Rha

**Affiliations:** 1 Department of Radiology, Uijeongbu St. Mary’s Hospital, College of Medicine, The Catholic University of Korea, Seoul 06591, Republic of Korea; radlsl@catholic.ac.kr; 2 Department of Radiology, Seoul St. Mary’s Hospital, College of Medicine, The Catholic University of Korea, Seoul 06951, Republic of Korea

**Keywords:** endometrial cancer, FIGO staging, prognostic factors, survival analysis, histological subtypes, lymphovascular space invasion

## Abstract

**Background/Objectives**: We comparatively evaluated the prognostic performance of the 2009 and 2023 International Federation of Gynecology and Obstetrics (FIGO) staging systems for early-stage endometrial cancer based on histological subtype stratification. **Methods**: A retrospective cohort of 472 patients with FIGO 2009 stage I–II between 2004 and 2019 was analyzed. Patients were restaged using both systems. Overall survival (OS) and recurrence-free survival (RFS) were determined according to histopathological aggressiveness. Kaplan–Meier survival analysis with log-rank testing compared the performance of the systems. Cox proportional hazards regression identified independent prognostic factors. A hypothetical modification of the FIGO 2023 system was evaluated for aggressive subtypes. **Results**: In all, 388 patients had nonaggressive histology, and 84 patients had aggressive histology. For cases of nonaggressive histology, FIGO 2023 demonstrated superior prognostic discrimination for OS and RFS (*p* < 0.05), whereas FIGO 2009 showed significant stratification for OS (*p* < 0.001) but not RFS (*p* = 0.149). For cases of aggressive histology, FIGO 2009 showed significant stratification for RFS (*p* = 0.017) but not OS (*p* = 0.31), whereas FIGO 2023 showed no significant stratification for either endpoint. The hypothetical modification of the FIGO 2023 staging system showed significantly improved discrimination for RFS (*p* = 0.019) but not OS. Multivariate analysis identified age and lymphovascular space invasion as independent prognostic factors in nonaggressive cancers, whereas cervical stromal involvement was significant in aggressive subtypes. **Conclusions**: The prognostic utility of the FIGO staging system is histology dependent. Although FIGO 2023 offers enhanced risk stratification for nonaggressive endometrial cancers, its discriminatory power for aggressive subtypes remains limited, indicating the need for histology-specific refinements of future staging frameworks.

## Simple Summary

Although endometrial cancer is often diagnosed at an early-stage, substantial heterogeneity exists in the recurrence risk among patients. We evaluated the comparative prognostic performance of two staging systems—the 2009 International Federation of Gynecology and Obstetrics (FIGO) staging system and revised 2023 FIGO classification—to determine their respective abilities to predict patient outcomes across different histological subtypes. We conducted a retrospective analysis of 472 patients with early-stage disease. Our findings demonstrated that the 2023 system exhibited superior prognostic discrimination for nonaggressive histological subtypes, whereas the 2009 system showed better predictive capacity for aggressive tumor types. Additionally, we evaluated a hypothetical modification of the FIGO 2023 staging system, which demonstrated enhanced prediction of disease recurrence in high-risk cases. These results support the implementation of histology-specific staging approaches and highlight the need for further refinement in aggressive tumor stratification.

## 1. Introduction

The International Federation of Gynecology and Obstetrics (FIGO) staging system serves as the cornerstone for endometrial cancer classification and treatment planning. The 2009 staging system established an anatomically based framework, primarily relying on myometrial invasion depth as the principal staging determinant [1]. However, many studies have revealed significant limitations of this traditional approach, particularly its inability to adequately reflect the biological heterogeneity and diverse clinical behaviors observed across different endometrial cancer subtypes. Consequently, patients with identical anatomical stages exhibit markedly different prognoses, highlighting the discordance between anatomical staging and tumor biology [2,3,4]. This limitation manifests clinically in cases where deep myometrial invasion coexists with favorable histological features, potentially resulting in overstaging, while minimal invasion accompanied by aggressive histological subtypes may lead to understaging and suboptimal treatment stratification [5,6].

To address these recognized deficiencies, the FIGO introduced a comprehensive revision of the endometrial cancer staging system in 2023, representing a paradigm shift toward a more biologically informed classification approach [5,7]. The FIGO 2023 system integrates multiple prognostic variables, including histological subtypes, extent of lymphovascular space invasion (LVSI), and molecular genetic alterations, such as POLE mutations and p53 abnormalities [8]. This multifaceted strategy has resulted in more detailed staging distinctions, especially in early-stage disease, where classification relies on the intricate interactions between myometrial invasion depth, histological features, and LVSI status [5,9].

Despite its theoretical advantages, the implementation of the FIGO 2023 system has been the subject of considerable debate [10,11,12,13]. Critics cite concerns about the increased complexity of the new system, potential reproducibility challenges, and inclusion of prognostic variables that lack sufficient validation in large studies. These questions are further amplified by uncertainties regarding the practical applicability of FIGO 2023 across diverse healthcare settings and its impact on clinical workflows. Additionally, recent comparative studies of FIGO 2009 and 2023 have reported mixed findings; some have demonstrated improved prognostic discrimination with the new system, while others showed no meaningful clinical benefits [14,15,16,17,18]. This heterogeneity underscores the need for additional validation studies across diverse patient cohorts to establish the clinical utility and practical feasibility of the revised staging criteria.

Given these considerations, we aimed to evaluate the prognostic performance of the FIGO 2023 staging system in a cohort of patients with early-stage endometrial cancer (stages I–II) from a single institution. Through a comparative analysis of survival outcomes between the FIGO 2009 and 2023 systems, we sought to determine whether integrating histological subtypes, LVSI status, and myometrial invasion offers superior prognostic stratification to the traditional anatomy-based approach. Additionally, we explored a potential modification of the FIGO 2023 staging system for aggressive tumors to determine whether further anatomical sub-classification can enhance prognostic discrimination. Our investigation builds upon recent analyses of the FIGO 2023 schema [9,19], providing a histology-stratified perspective on its prognostic performance while introducing an exploratory approach to refining staging for high-risk diseases.

## 2. Materials and Methods

### 2.1. Study Population

**Figure 1:**
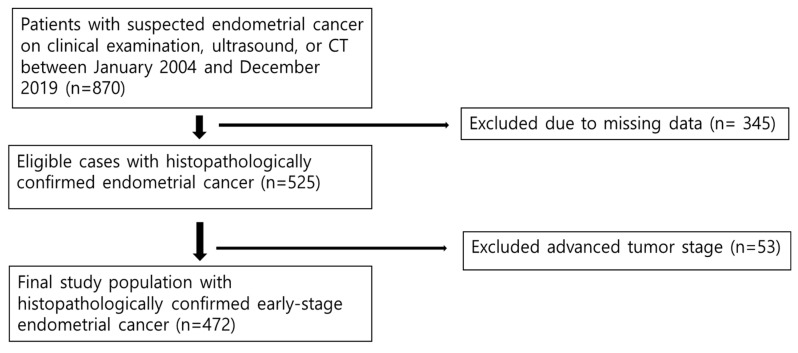
Flow diagram of the study population.

This retrospective cohort study was approved by the Institutional Review Board, which waived the requirement for informed consent. Between January 2004 and December 2019, we systematically identified 870 patients with suspected endometrial cancer who underwent evaluation and treatment at our institution. The inclusion criteria were as follows: (1) pathologically confirmed endometrial cancer, (2) availability of complete clinical and histopathological data required for staging assessment, and (3) adequate electronic medical record documentation. Patients were excluded if they lacked sufficient pathological documentation that precluded accurate staging assessments or if they had inconclusive pathological diagnoses (n = 345). After applying these criteria, 525 patients with confirmed endometrial cancer were identified. For this comparative staging analysis, we further restricted the cohort to those with early-stage disease (FIGO 2009 stages I–II), resulting in the exclusion of 53 patients with advanced-stage disease (FIGO 2009 stage III, n = 44; stage IV, n = 9). The final study cohort comprised 472 patients with early-stage endometrial cancer (Figure 1).

### 2.2. Histopathological Assessment

All included patients underwent primary surgical treatment, and histopathological evaluations were performed independently by three board-certified pathologists with subspecialty expertise in gynecological pathology to ensure diagnostic accuracy and consistency. Standardized pathological parameters were systematically evaluated for each case, including myometrial invasion depth (categorized as no invasion, <50% invasion, or ≥50% invasion), histological subtype, presence and extent of LVSI, and cervical stromal invasion (CSI). LVSI was assessed using hematoxylin and eosin staining and classified as present or absent [20]. CSI was defined as a direct extension of the tumor into the cervical stroma. Histological subtypes were classified as aggressive (grade 3 endometrioid adenocarcinoma, serous carcinoma, clear cell carcinoma, and carcinosarcoma) or nonaggressive (grade 1–2 endometrioid adenocarcinoma), based on established prognostic classifications.

### 2.3. Staging Systems and Data Collection

Data of comprehensive demographic and clinicopathological variables, including age, menopausal status, tumor size, and recurrence patterns, were systematically extracted from electronic medical records. All patients were retrospectively restaged according to the FIGO 2009 and 2023 staging criteria to enable a direct comparative analysis. Under the FIGO 2009 system, early-stage endometrial carcinoma is defined purely by tumor extent: Stage I tumors are confined to the uterus (IA for <50% myometrial invasion; IB for ≥50% invasion), and stage II indicates cervical stromal involvement [1]. In the FIGO 2023 system, staging incorporates tumor histology and LVSI [5]. Low-grade (nonaggressive) histological types were staged as IA1 (tumor limited to the endometrium with no myometrial invasion), IA2 (invasion < 50% without substantial LVSI), or IB (invasion ≥ 50% without substantial LVSI). If substantial LVSI was present in a low-grade tumor, the patient was upstaged to IIB, and cervical stromal involvement without substantial LVSI was classified as IIA. High-grade (aggressive) histological types were designated as IC when the tumor was confined to the endometrium (no myometrial invasion) and as IIC when any myometrial and/or CSI existed [5]. Molecular features (such as POLE mutations and p53 status) introduced in the FIGO 2023 schema were not applied in our restaging owing to the lack of available molecular data; consequently, all patients were staged exclusively according to anatomical and histopathological criteria.

The primary analytical objective of this comparative study was to evaluate the prognostic discriminatory capacity of the histological dichotomous classification, which represents a fundamental modification of the FIGO 2023 system. For this assessment, patients were stratified into the following two histological groups: nonaggressive and aggressive subtypes. This stratification approach enabled an unbiased comparison of the prognostic classification performance between the two staging systems for histologically homogeneous subgroups. As a secondary, exploratory objective, we developed a hypothetical modification of the FIGO 2023 system to investigate potential refinements in the staging of aggressive histological subtypes. This exploratory schema, based on the hypothesis that anatomical progression remains clinically relevant within aggressive histological subtypes, introduced anatomical progression-based substaging for patients with aggressive histological types: stage IC for tumors confined to the endometrium without myometrial invasion, stage IIC1 for tumors with <50% myometrial invasion, stage IIC2 for tumors with >50% myometrial invasion, and stage IIC3 for tumors with CSI. This modified classification system was designed to test the hypothesis that enhanced substaging could improve prognostic stratification within the aggressive tumor subtype category. Of note, this modified schema was entirely hypothetical, does not constitute part of any official FIGO staging system, and was employed exclusively for exploratory analysis in this study.

### 2.4. Outcome Definitions

The primary endpoints were overall survival (OS) and recurrence-free survival (RFS). OS was defined as the time from initial diagnosis to death from any cause or the date of the last clinical follow-up for patients who remain alive. RFS was defined as the time from primary surgery to the first documented recurrence or the last follow-up date for patients without recurrence. Disease recurrence was defined using standardized criteria: biopsy-confirmed disease recurrence or new focal findings on postoperative computed tomography (CT) or magnetic resonance imaging (MRI) suggestive of malignancy, as confirmed by multidisciplinary team review. Patients with postoperative imaging abnormalities confirmed to be pathologically or clinically benign were considered recurrence-free. Recurrence patterns were systematically classified as locoregional or distant recurrence, based on the anatomical site of the first recurrence. Locoregional recurrence was defined as occurrence within the pelvis, such as vaginal and pelvic recurrence involving pelvic lymph nodes and local extension to the rectum and bladder. Distant recurrence included extrapelvic disease, including peritoneal carcinomatosis; omental metastasis; distant metastases to the lung, liver, bone, and brain; and extrapelvic lymph node involvement.

### 2.5. Statistical Analysis

Descriptive statistics were calculated to summarize patient and tumor characteristics. Categorical variables were presented as frequencies and percentages, while continuous variables were reported as means ± standard deviations and medians with interquartile ranges. The primary analytical approach used Kaplan–Meier survival analysis with log-rank testing to evaluate and compare OS and RFS distributions across staging systems and histological subgroups. Survival curves were constructed for each staging system within histological subgroups, and statistical significance was assessed using the log-rank test. To identify independent prognostic factors, we used univariate and multivariate Cox proportional hazards regression models, stratified by histological aggressiveness. Variables that achieved statistical significance (*p* < 0.05) in the univariate analysis were incorporated into the multivariate models using the forward selection methodology. All statistical tests were two-sided, with statistical significance set at *p* <0.05. Statistical analyses, including Kaplan–Meier survival analysis, hazard ratio (HR) calculation, and log-rank tests for survival curve comparison, were conducted using MedCalc software (version 23.3.2; MedCalc Software Ltd., Ostend, Belgium). Additional analyses were performed using SAS software (version 9.4, SAS Institute, Cary, NC, USA) and R software (version 4.1.2; R Foundation for Statistical Computing, Vienna, Austria).

## 3. Results

### 3.1. Baseline Characteristics

A total of 472 patients were enrolled in this study. Table 1 summarizes the clinicopathological characteristics of the patients. The mean patient age was 55.0 ± 9.8 years, with a median of 55 years (interquartile range: 49–61 years). Most patients (64%) were postmenopausal, and the mean maximum tumor diameter was 3.1 ± 2.3 cm. According to the FIGO 2009 staging system, 336 patients (71.2%) were classified as stage IA, 96 (20.3%) were classified as stage IB, and 40 (8.5%) were classified as stage II. The FIGO 2023 staging system demonstrated a more granular distribution across substages, with stages IA1 (24.4%) and IA2 (32.4%) representing the most frequent categories. The study sample predominantly consisted of patients with nonaggressive histological subtypes, with 388 patients (82.2%) having grade 1–2 endometrioid adenocarcinoma, and 84 patients (17.8%) had aggressive histological subtypes. During the follow-up period, 47 patients (10%) experienced disease recurrence, with distant recurrence patterns being predominant (76.6% of recurrences), and 48 patients (10.2%) had died.

### 3.2. Survival Outcomes by Histological Subgroup (FIGO 2009 vs. 2023)

**Figure 2:**
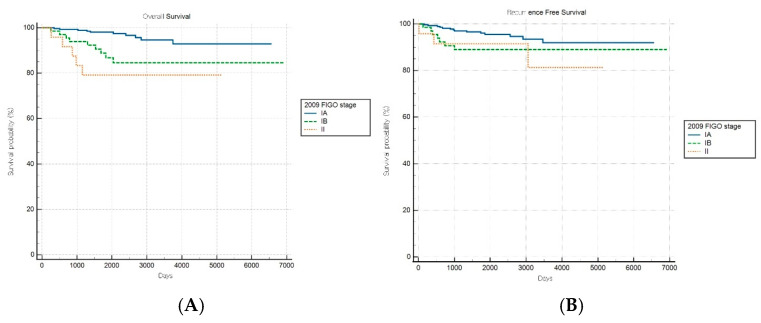
Kaplan–Meier survival curves for (**A**) overall survival (log-rank *p* < 0.001) and (**B**) recurrence-free survival (log-rank *p* = 0.149) for patients with nonaggressive histology according to the FIGO 2009 staging system.

Comparative survival analyses stratified by histopathological aggressiveness revealed differential prognostic performance between the FIGO staging systems across the nonaggressive and aggressive endometrial cancer subgroups. For patients with nonaggressive histological subtypes, the FIGO 2009 staging system demonstrated significant prognostic discrimination for OS (log-rank *p* < 0.001) but not for RFS (log-rank *p* = 0.149) (Figure 2A,B). Patients with stage IA disease exhibited significantly better OS than those with stages IB (HR: 3.61, 95% CI: 1.28–10.18) and II (HR: 5.72, 95% CI: 1.12–29.22), while no significant survival difference was observed between stages IB and II (HR: 1.58, 95% CI: 0.26–9.75).

**Figure 3:**
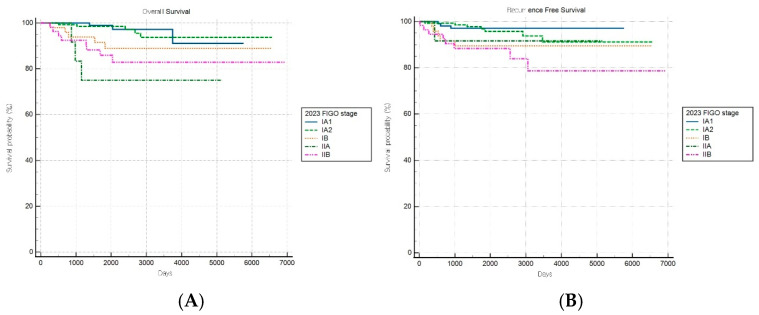
Kaplan–Meier survival curves for (**A**) overall survival (log-rank *p* < 0.001) and (**B**) recurrence-free survival (log-rank *p* = 0.039) for patients with nonaggressive histology according to the FIGO 2023 staging system.

Conversely, the FIGO 2023 system achieved significant prognostic stratification for both OS and RFS in patients with nonaggressive histological subtypes (log-rank *p* < 0.001 and *p* = 0.039, respectively; Figure 3A,B). A stepwise increase in HR was observed with advancing stage, with an elevated mortality risk noted in intermediate stages (e.g., HRs of 3.30 [95% CI: 0.91–12.00] and 8.52 [95% CI: 0.85–85.92] for specific stages). Although the HR for one intermediate stage appeared numerically higher than that for a more advanced stage, the pairwise differences in OS between these stages were not statistically significant, suggesting variability within stage subgroups and underscoring the need for further refinement and validation of the stage definitions.

**Figure 4:**
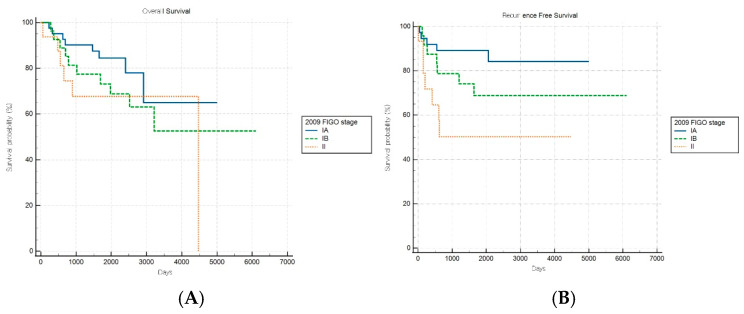
Kaplan–Meier survival curves for (**A**) overall survival log-rank (*p* = 0.31) and (**B**) recurrence-free survival (log-rank *p* = 0.017) for patients with aggressive histology according to the FIGO 2009 staging system.

For patients with aggressive histological subtypes, the FIGO 2009 staging system maintained significant prognostic discrimination for RFS (log-rank *p* = 0.017) but failed to achieve significant stratification for OS (log-rank *p* = 0.31) (Figure 4A,B). Notably, one stage subgroup exhibited a significantly lower risk of recurrence than the reference group (HR: 0.21, 95% CI: 0.06–0.78), suggesting that the 2009 system retains some prognostic value in predicting recurrence outcomes within aggressive tumor types.

**Figure 5:**
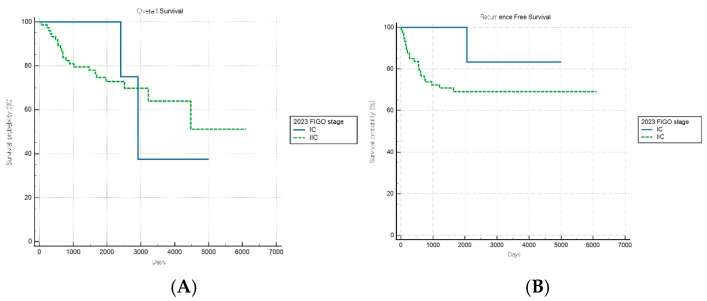
Kaplan–Meier survival curves for (**A**) overall survival (log-rank *p* = 0.490) and (**B**) recurrence-free survival (log-rank *p* = 0.176) for patients with aggressive histology according to the FIGO 2023 staging system.

In contrast, the FIGO 2023 system demonstrated no significant stratification for either survival endpoint (both *p* > 0.05) in the aggressive subgroup, suggesting a diminished prognostic utility in this high-risk population (Figure 5A,B). These findings indicate that, while FIGO 2023 staging provides enhanced discrimination for low-risk cancers, it may inadequately capture the biological heterogeneity within aggressive endometrial cancer subtypes.

**Figure 6:**
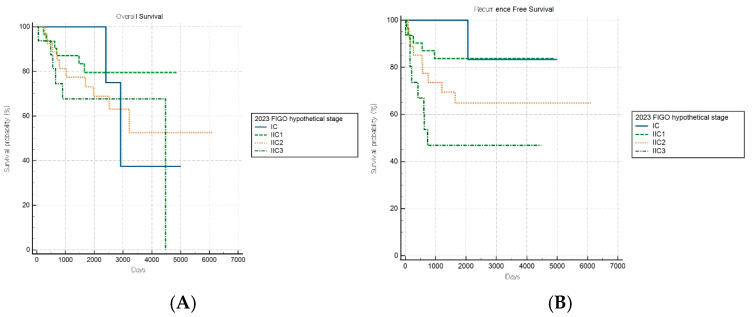
Kaplan–Meier survival curves for (**A**) overall survival (log-rank *p* = 0.497) and (**B**) recurrence-free survival (log-rank *p* = 0.019) for patients with aggressive histology according to the hypothetical modification of the FIGO 2023 staging system.

Of note, the hypothetical modification of the FIGO 2023 staging system demonstrated improved discriminatory capacity for RFS (log-rank *p* = 0.019) within the aggressive subgroup, while OS stratification remained nonsignificant (*p* = 0.497) (Figure 6A,B). This selective improvement in recurrence risk stratification suggests that introducing additional anatomical substages may enhance the risk discrimination for aggressive tumors. However, this modified schema was experimental and requires further validation.

Overall, our findings demonstrate that the relative prognostic performance of the FIGO staging system is dependent on histology. The FIGO 2023 system provides notably improved risk stratification for nonaggressive (low-grade) endometrial cancers but shows substantially diminished prognostic utility for aggressive (high-grade) subtypes. Conversely, the FIGO 2009 system retains some discriminatory capacity for aggressive cancers, particularly regarding recurrence risk. The improved RFS stratification observed with the exploratory FIGO 2023 modification in aggressive cancers warrants further investigation and remains unvalidated. The consistent lack of OS stratification across all staging systems for aggressive histological subtypes highlights the need for additional prognostic factors beyond anatomical staging in this patient population.

**Figure 7:**
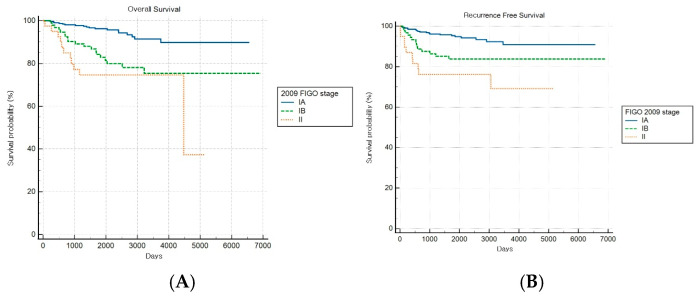
Kaplan–Meier survival curves for (**A**) overall survival (log-rank *p* < 0.001) and (**B**) recurrence-free survival (log-rank *p* < 0.001) for patients with combined histology according to the FIGO 2009 staging system.

Among patients with combined subtypes, OS and RFS analyses based on the FIGO 2009 staging system revealed a statistically significant stage-based stratification across stages (both log-rank *p* < 0.001) (Figure 7A,B). However, no significant difference in OS outcomes was observed between stages IB and II (HR: 1.49, 95% CI: 0.47–4.76).

**Figure 8:**
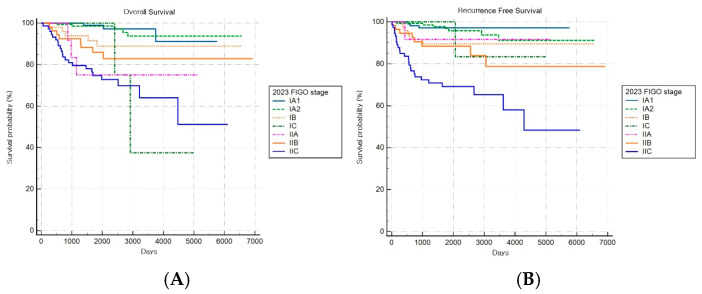
Kaplan–Meier survival curves for (**A**) overall survival (log-rank *p* < 0.001) and (**B**) recurrence-free survival (log-rank *p* < 0.001) according to the revised FIGO 2023 staging system.

The FIGO 2023 staging system demonstrated a statistically significant stage-based stratification for both OS and RFS across stages (both log-rank *p* < 0.001) (Figure 8A,B). Significant increments in mortality were observed among patients with stages IB, IIA, and IIC disease compared to patients with stage IA1 disease, while those with stage IA2 disease did not show significant changes.

### 3.3. Prognostic Factors Stratified by Histological Subtype (Nonaggressive vs. Aggressive)

Table 2 and Table 3 present the results of univariate and multivariate Cox regression analyses for RFS and OS, stratified by histological aggressiveness. For patients with nonaggressive histological subtypes, two independent prognostic factors for RFS were identified: advancing age (HR: 1.04; 95% CI: 1.00–1.09; *p* = 0.035) and presence of LVSI (HR: 2.80; 95% CI: 1.34–5.85; *p* = 0.006). Conversely, for patients with aggressive histological subtypes, CSI emerged as the sole significant prognostic factor for RFS (HR: 2.65; 95% CI: 1.31–5.34; *p* = 0.007) (Table 2).

Regarding the prognostic factors for OS, stratified analysis yielded histology-specific results. For patients with nonaggressive histological subtypes, univariate analysis revealed four significant factors associated with reduced OS: advanced age (HR: 1.08; 95% CI: 1.03–1.12; *p* = 0.001), presence of LVSI (HR: 3.57; 95% CI: 1.7–7.47; *p* = 0.001), deep myometrial invasion (HR: 2.85; 95% CI: 1.17–6.92; *p* = 0.02), and presence of CSI (HR: 3.2; 95% CI: 1.3–7.88; *p* = 0.01). Subsequent multivariate analysis identified two independent prognostic factors: advanced age (HR: 1.07; 95% CI: 1.01–1.13; *p* = 0.01) and presence of LVSI (HR: 2.39; 95% CI: 1.00–5.69; *p* = 0.049). For patients with aggressive histological subtypes, univariate analysis identified two significant prognostic factors for OS: advanced age (HR: 1.05; 95% CI: 1.01–1.08; *p* = 0.008) and presence of CSI (HR: 2.18; 95% CI: 1.06–4.49; *p* = 0.03). In the multivariate analysis, only age remained independently associated with reduced OS (HR: 1.05; 95% CI: 1.01–1.08; *p* = 0.015) (Table 3).

These analyses revealed distinct histology-specific prognostic patterns: nonaggressive endometrial cancers demonstrated sensitivity to both LVSI and patient age for both survival endpoints, while aggressive cancers showed predominant dependence on anatomical extension (CSI) for recurrence risk and patient age for OS. These distinct prognostic factor profiles between the histological subgroups underscore the importance of conducting separate analyses for nonaggressive and aggressive endometrial cancers.

## 4. Discussion

The most significant advancements in the FIGO 2023 staging system for endometrial cancer include the introduction of categorization based on molecular and histopathological data, including distinguishing between nonaggressive and aggressive subtypes, introducing LVSI as a staging criterion, and redefining the absence of myometrial invasion as a distinct prognostic factor [10,21,22,23]. Under this schema, tumors are classified into nonaggressive and aggressive groups, with separate thresholds applied for invasion depth and LVSI in each category. For nonaggressive subtypes, the system employs a refined approach to myometrial invasion stratification: tumors lacking invasion are classified as IA1, while those with invasion are further subdivided into IA2 (<50% myometrial invasion) and IB (≥50% invasion). A critical feature of this system is the upstaging to IIB when LVSI is present in the nonaggressive subtype, regardless of invasion depth, thereby reflecting LVSI’s established importance as an independent prognostic indicator, as demonstrated in recent studies and guidelines [10,24]. In contrast, for aggressive subtypes, a simplified staging approach was employed: tumors without myometrial invasion are designated as IC, whereas those with any degree of myometrial or CSI are collectively classified as IIC. This structure aims to enhance the precision of prognostic prediction and clinical utility by better accounting for the biological heterogeneity of endometrial tumors [10,21]. However, the 2009 FIGO system relies exclusively on myometrial invasion depth and does not incorporate histological subtypes. This fundamental difference could introduce analytical bias when comparing the prognostic capabilities of the two systems. To address this challenge, we stratified patients by histological data before comparison, thereby ensuring that the prognostic performance of each system was assessed within homogeneous histological groups.

Our comparative analysis revealed differential prognostic performances between the two staging systems across histological subtypes. For nonaggressive tumors, the 2009 FIGO system provided significant prognostic discrimination for OS but not for RFS. Conversely, the FIGO 2023 system achieved a consistent prognostic separation for both endpoints, suggesting improved risk stratification. Intermediate stage categories under the 2023 system showed elevated HR (e.g., IB—HR: 3.30 [95% CI: 0.91–12.00]; IIA—HR: 8.52 [95% CI: 0.85–85.92]), although not all comparisons reached statistical significance. These findings indicate a trend toward better prognostic alignment under the revised framework, even in the absence of strong pairwise significance. These results align with the findings of Matsuo et al., who documented a wider OS difference and clearer outcome separation under the new system as well as with the results of a recent international analysis that demonstrated improved 5-year RFS for stage I patients under the FIGO 2023 [9,19]. This enhanced discrimination underscores the clinical value of integrating LVSI into staging, thereby enabling finer risk stratification and supporting individualized treatment planning. The demonstration of LVSI as an independent prognostic factor for low-grade endometrial cancer in this cohort aligns with the results of previous studies [10,25,26]. Therefore, its incorporation into FIGO 2023 facilitates more precise decisions regarding adjuvant therapy and fertility-sparing options, which are particularly relevant given the increasing incidence of endometrial cancer in premenopausal populations. Contemporary clinical guidelines have similarly affirmed the independent prognostic value of LVSI [10,21,25,27]. The revised staging system also has important clinical implications for fertility-sparing management. In carefully selected young patients with low-grade endometrioid histology confined to the endometrium (stage IA1) and negative LVSI, accurate staging is critical for determining eligibility for conservative hormonal therapy [28]. The enhanced prognostic stratification provided by the new FIGO classification facilitates more personalized risk assessment and safer fertility-preserving approaches.

An additional noteworthy modification of the 2023 FIGO staging system is the subdivision of stage I into IA1 and IA2 based on the presence or absence of myometrial invasion. However, prior studies have reported comparable survival outcomes between patients with the IA1 and IA2 disease subtypes [19]. This observed prognostic similarity raises questions regarding the clinical utility of this refined stratification, particularly for noninvasive tumors, suggesting that the separation between endometrium-confined tumors and those with minimal myometrial invasion may represent an over classification within the revised staging framework. Further validation is required to determine whether this subdivision provides meaningful prognostic discrimination.

In contrast to the improved performance observed in nonaggressive tumors, both staging systems showed notable limitations when applied to aggressive histological subtypes. While FIGO 2009 exhibited significant RFS stratification (*p* = 0.017) but not OS stratification (*p* = 0.310), FIGO 2023 failed to significantly stratify either outcome (both *p* > 0.05), indicating a diminished prognostic capacity within this high-risk subgroup. To explore potential improvements in risk stratification for aggressive tumors, we evaluated a hypothetical modification of FIGO 2023 that subdivided stage IIC by myometrial invasion depth and CSI: IIC1 (<50% myometrial invasion), IIC2 (≥50%), and IIC3 (presence of CSI). This revised schema improved RFS discrimination (*p* = 0.02), although OS stratification remained nonsignificant. These findings suggest that the current single IIC category may obscure biologically relevant heterogeneity and that increased anatomical granularity could optimize recurrence risk prediction for aggressive histological subtypes [18].

Multivariate analysis revealed histology-specific prognostic determinants, further supporting the rationale for differentiated staging approaches. Cox regression analysis identified distinct prognostic profiles based on histological findings. For patients with nonaggressive tumors, older age and LVSI independently predicted both OS and RFS, highlighting the sensitivity of low-grade disease outcomes to these pathological variables. Conversely, CSI was the primary predictor of recurrence risk in patients with aggressive tumors, while age was the sole independent factor for OS [29]. These divergent patterns support the need for histology-tailored staging criteria and justify the exclusion of LVSI as a staging determinant for aggressive subtypes under FIGO 2023 [18]. However, other studies have reported that extensive LVSI correlates with decreased locoregional and distant disease-free survival, even in aggressive histological types, suggesting that LVSI may provide prognostic information not captured by current staging criteria [30,31,32,33]. This discrepancy emphasizes the need for further multicenter validation and refinement of FIGO 2023, particularly for aggressive subgroups. Furthermore, combining myometrial and cervical invasions into a single IIC category may obscure distinct recurrence or metastasis risk patterns in aggressive cases [34]. Therefore, future improvements to the staging system should incorporate more detailed differentiation of invasion extent and comprehensive reassessment of key pathological parameters [35].

Another important consideration is the role of imaging in preoperative staging of the disease. Imaging remains central to treatment planning and surgical decision making. Transvaginal ultrasound (TVS) and MRI demonstrate comparable diagnostic performance when all patients with endometrial cancer are considered; however, for patients with low-grade tumors, MRI provides superior specificity to TVS [36]. Recent advances in preoperative MRI-based radiomics analyses have further enhanced the role of MRI, showing strong predictive performance for tumor grading, deep myometrial invasion, LVSI, and nodal metastasis in patients with endometrial carcinoma [37]. These findings highlight that radiomics-augmented MRI not only improves anatomical assessment but also provides quantitative biomarkers that may refine risk stratification.

This study has some limitations that warrant consideration. First, its retrospective design precluded the inclusion of molecular classification, a critical component of the FIGO 2023 system. Given that molecular subtypes are crucial for prognosis and treatment planning, their absence may compromise staging accuracy [38]. Second, restricting the cohort to cases with complete data may have introduced selection bias; specifically, approximately 40% of the patients were excluded during initial screening because of missing histological or invasion data, predominantly from earlier years (2004–2007) when reporting standards differed. Nonetheless, our final sample included patients with reliable and standardized data, thereby enhancing the internal validity of the comparative staging analysis. Third, the newly added IA3 stage in FIGO 2023 could not be evaluated in our analysis because of the absence of qualifying cases. Fourth, the study population was limited to surgically treated patients with early-stage disease at a single institution, constraining its generalizability to advanced-stage cases, nonsurgical management, and diverse populations across different settings. Fifth, the small number of events in the aggressive subgroup may have reduced statistical power, thus necessitating a cautious interpretation of the hazard ratio estimates. Seventh, detailed data on adjuvant treatments (e.g., radiation, chemotherapy) were unavailable in our dataset, precluding the analysis of how postsurgical therapy might have influenced the outcomes. Consequently, variations in adjuvant treatment strategies could represent confounding factors when comparing survival between staging systems. Furthermore, the assessment of LVSI was based exclusively on HE staining, which was the standard of care during the study period (2004–2019). We acknowledge that the use of immunohistochemical markers, such as D2-40, could have improved the sensitivity of LVSI detection. This represents a potential limitation, although the review by specialist gynecological pathologists likely minimized misclassification. Finally, this study involved the use of a hypothetical staging framework proposed by the authors to operationalize the 2023 classification system. Although this approach was necessary to enable a comparative evaluation with the 2009 system, it inevitably introduced a degree of author-defined bias. We sought to mitigate this by performing sensitivity analyses without hypothetical staging, which yielded results consistent with the main findings. To address these limitations and improve the external validity of the FIGO 2023 staging system, multicenter prospective studies encompassing all disease stages and treatment modalities are required.

## 5. Conclusions

The FIGO 2023 system for endometrial cancer marks a significant advancement over the 2009 classification by incorporating histopathological subtypes and the prognostic significance of LVSI. This integration enhances risk stratification, especially in early-stage disease; however, studies of long-term clinical outcomes are needed to validate this system and support its clinical utility.

## Figures and Tables

**Table 1:** Demographics and clinical characteristics of the study population.

Characteristics	n = 472
**Age (years)**	
Mean ± SD	55 ± 9.8
Median (IQR)	55 (49, 61)
**Maximum tumor diameter (cm)**	
Mean ± SD	3.1 ± 2.3
Median (IQR)	2.9 (1.7, 4.5)
**Postmenopausal**	302 (64)
**FIGO stage 2009**	
IA	336 (71.2)
IB	96 (20.3)
II	40 (8.5)
**FIGO stage 2023**	
IA1	115 (24.4)
IA2	153 (32.4)
IB	50 (10.6)
IC	10 (2.1)
IIA	12 (2.5)
IIB	58 (12.3)
IIC	74 (15.7)
**Endometrial cancer subtype**	
Endometrioid adenocarcinoma (grade 1–2)	388 (82.2)
Endometrioid adenocarcinoma (grade 3)	45 (9.6)
Mucinous carcinoma	3 (0.6)
Serous carcinoma	15 (3.2)
Clear cell carcinoma	4 (0.8)
Mixed carcinoma	2 (0.4)
Carcinosarcoma	15 (3.2)
**Recurrence**	
No	425 (90)
Yes	47 (10)
**Pattern of recurrence**	
Locoregional	11 (23.4)
Distant	36 (76.6)
**Death**	
No	424 (89.8)
Yes	48 (10.2)

Values are presented as numbers (percentages) for categorical variables and as mean ± SD and median (IQR) for continuous variables.

**Table 2:** Cox proportional hazard model univariate and multivariate analyses for recurrence-free survival stratified by histological aggressiveness.

	Nonaggressive Group				Aggressive Group			
Variables	Univariate		Multivariate		Univariate		Multivariate	
	HR (95% CI)	*p*-Value	HR (95% CI)	*p*-Value	HR (95% CI)	*p*-Value	HR (95% CI)	*p*-Value
Age	1.044 (1.003–1.086)	0.035	1.036 (0.984–1.092)	0.18	1.019 (0.985–1.054)	0.282	1.018 (0.982–1.056)	0.332
Menopausal status	1.701 (0.783–3.695)	0.179	0.972 (0.370–2.555)	0.955	0.809 (0.379–1.729)	0.585	0.804 (0.343–1.886)	0.616
LVSI	2.800 (1.341–5.845)	0.006	2.181 (0.934–5.094)	0.072	0.880 (0.452–1.712)	0.707	0.587 (0.286–1.207)	0.148
Myometrial invasion depth								
No invasion	(reference)		(reference)		(reference)		(reference)	
<50% invasion	1.055 (0.415–2.682)	0.91	0.884 (0.344–2.268)	0.798	1.242 (0.264–5.849)	0.784	1.377 (0.285–6.662)	0.691
≥50% invasion	2.223 (0.874–5.652)	0.093	1.129 (0.389–3.277)	0.823	3.118 (0.738–13.180)	0.122	3.523 (0.761–16.315)	0.107
CSI	2.401 (0.922–6.254)	0.073	1.508 (0.526–4.322)	0.445	2.646 (1.311–5.339)	0.007	2.126 (0.999–4.524)	0.0503

HR, hazard ratio; CI, confidence interval; LVSI, lymphovascular space invasion; CSI, cervical stromal invasion.

**Table 3:** Cox proportional hazard model univariate and multivariate analyses for overall survival stratified by histological aggressiveness.

	Nonaggressive Group				Aggressive Group			
Variables	Univariate		Multivariate		Univariate		Multivariate	
	HR (95% CI)	*p*-Value	HR (95% CI)	*p*-Value	HR (95%CI)	*p*-Value	HR (95% CI)	*p*-Value
Age	1.075 (1.031–1.120)	0.001	1.069 (1.014–1.127)	0.014	1.047 (1.012–1.083)	0.008	1.045 (1.009–1.084)	0.015
Menopausal status	1.823 (0.808–4.116)	0.148	0.699 (0.260–1.881)	0.478	1.352 (0.580–3.152)	0.486	1.166 (0.474–2.865)	0.739
LVSI	3.566 (1.702–7.471)	0.001	2.391 (1.004–5.691)	0.049	0.934 (0.476–1.833)	0.843	0.690 (0.327–1.457)	0.33
Myometrial invasion depth								
No invasion	(reference)		(reference)		(reference)		(reference)	
<50% invasion	0.520 (0.175–1.548)	0.24	0.421 (0.140–1.262)	0.123	1.246 (0.264–5.871)	0.781	1.440 (0.295–7.034)	0.653
≥50% invasion	2.845 (1.169–6.920)	0.021	1.198 (0.432–3.323)	0.729	2.693 (0.636–11.411)	0.179	2.644 (0.563–12.422)	0.218
CSI	3.203 (1.301–7.883)	0.011	1.631 (0.595–4.474)	0.342	2.182 (1.062–4.487)	0.034	2.108 (0.982–4.523)	0.056

HR, hazard ratio; CI, confidence interval; LVSI, lymphovascular space invasion; CSI, cervical stromal invasion.

## Data Availability

The datasets generated and/or analyzed in this study are available from the corresponding author upon reasonable request.

## Abbreviations

The following abbreviations are used in this manuscript:

FIGO	International Federation of Gynecology and Obstetrics
OS	Overall Survival
RFS	Recurrence-Free Survival
LVSI	Lymphovascular Space Invasion
CSI	Cervical Stromal Invasion
CT	Computed Tomography
MRI	Magnetic Resonance Imaging
HR	Hazard Ratio
CI	Confidence Interval
SD	Standard Deviation
IQR	Interquartile Range
